# Analysis of inappropriate prophylactic use of proton pump inhibitors during the perioperative period: an observational study

**DOI:** 10.1186/s13741-024-00376-w

**Published:** 2024-03-14

**Authors:** Pengpeng Liu, Guangyao Li, Qian Wu, Mei Han, Chao Zhang

**Affiliations:** 1 Department of Pharmacy, Beijing Tongren Hospital, Capital Medical University, Beijing, 100730 China; 2 Department of Neurology, Beijing Tongren Hospital, Capital Medical University, Beijing, China; 3 Evidence-Based Medicine Center, Beijing University of Chinese Medicine, Beijing, China

**Keywords:** Perioperative period, Stress-related mucosal disease, Proton pump inhibitors, Prophylaxis

## Abstract

**Background:**

The prevalence and characteristics of inappropriate use of proton pump inhibitors (PPIs) to prevent stress-related mucosal disease (SRMD) during the perioperative period and its associated factors are rarely reported. This study aimed to investigate the prevalence and characteristics of inappropriate prophylactic use of proton pump inhibitors (PPIs) during the perioperative period and identify its associated factors in a tertiary care and academic teaching hospital in China and to provide evidence for regulation authorities and pharmacists to take targeted measures to promote rational drug use.

**Methods:**

Inpatients who underwent surgical operations and received prophylactic use of PPIs from June 2022 to November 2022 were included in this retrospective study. The appropriateness of perioperative prophylactic use of PPIs was evaluated by clinical pharmacists. Associated factors with inappropriate perioperative prophylactic use of PPIs were analyzed by univariable and multivariable logistic regression.

**Results:**

Four-hundred seventy-two patients were finally included in this study, of which 131 (27.75%) patients had at least one problem with inappropriate perioperative prophylactic use of PPIs. The three most common problems were drug use without indication (52.0%), inappropriate usage and dosage (34.6%), and inappropriate duration of medication (6.7%). Multiple logistic regression analysis showed that oral dosage form of PPIs [*OR* = 18.301, 95% *CI* (7.497, 44.671), *p* < 0.001], discharge medication of PPIs [*OR* = 11.739, 95% *CI* (1.289, 106.886), *p* = 0.029], and junior doctors [*OR* = 9.167, 95% *CI* (3.459, 24.299), *p* < 0.001] were associated with more inappropriate prophylactic use of PPIs. Antithrombotics [*OR* = 0.313, 95% *CI* (0.136, 0.721), *p* = 0.006] and prolonged postoperative hospital stay (longer than 15 days) [*OR* = 0.262, 95% *CI* (0.072, 0.951), *p* = 0.042] were associated with less inappropriate prophylactic use of PPIs.

**Conclusions:**

The inappropriate prophylactic use of PPIs during the perioperative period is common. Regulation authorities and pharmacists should take more targeted measures to promote the rational prophylactic use of PPIs during the perioperative period.

**Supplementary Information:**

The online version contains supplementary material available at 10.1186/s13741-024-00376-w.

## Introduction

Stress-related mucosal disease (SRMD), also known as stress ulcer, refers to the acute gastrointestinal mucosal erosion, ulceration, and other lesions occurring in the body under various stress states such as severe trauma, complex surgery, and critical illness, which can lead to gastrointestinal bleeding or even perforation in severe cases (Bardou et al. 2015; Toews et al. 2018; Grube and May 2007). It can aggravate and worsen the degree of the original disease and increase the fatality rate (Bardou et al. 2015). Various difficult and complex surgeries are common stressors that induce SRMD (Li et al. 2022; Quenot et al. 2009). Prevention and treatment of perioperative SRMD can improve perioperative safety, shorten hospital stays, and reduce medical costs for high-risk surgical patients (Surgical Society of Chinese Medical Association 2015; Singh et al. 2016). Medications such as proton pump inhibitors (PPIs) and histamine-2 receptor antagonists (H_2_RAs) are commonly administered prophylactically to reduce the risk of SRMD (Clarke et al. 2022). PPIs, acting on H + /K + ATPase (the proton pump), inhibit the last channel of gastric acid secretion and have a good inhibitory effect on basal and food-stimulated acid secretions. Moreover, they have strong and longer-lasting inhibiting effects on gastric acid secretion and are safe and well tolerated (Helgadottir and Bjornsson 2019; Savarino et al. 2017; Ali et al. 2019). Currently, they are the preferred drugs for the clinical prevention and treatment of acid-related diseases (Bardou et al. 2015; Savarino et al. 2017; Alhazzani et al. 2018).

In recent years, PPIs have been widely used in the prevention of SRMD, especially in the perioperative period, and the problem of irrational drug use has become increasingly prominent (Chen et al. 2022; Savarino et al. 2018). The inappropriate prophylactic use of PPIs has not only increased the economic burden on patients, and caused the waste of medical resources, but has also raised the potential medical risks (Li et al. 2022). Therefore, the Chinese authorities have taken various measures to manage the inappropriate prophylactic use of PPIs and decrease medical costs. Studies have shown that 48.9–79% of surgical inpatients were inappropriately prescribed PPIs to prevent SRMD (Chen et al. 2022; Zhang et al. 2021; Bez et al. 2013). However, these studies were limited to specific single surgical departments such as general and hepatobiliary surgery and the analyses of pharmaceutical intervention. To date, research on the prevalence and characteristics of inappropriate prophylactic use of PPIs during the perioperative period and its associated factors is sparse.

Therefore, the primary objective of this study was to evaluate the appropriateness of perioperative prophylactic use of PPIs in inpatients. The second objective was to identify associated factors with the inappropriate prophylactic use of PPIs.

## Methods

### Setting and study design

This retrospective study was conducted in the Beijing Tongren Hospital affiliated with Capital Medical University, a 1759-bed tertiary care, teaching, and research institution. Patients who underwent surgical operations and received prophylactic use of PPIs during hospitalization from June 2022 to November 2022 were included in this study. Patients who were prescribed PPIs for the treatment of gastrointestinal diseases such as gastrointestinal hemorrhage, peptic ulcer, and gastroesophageal reflux disease were excluded. Patients with incomplete data were also excluded. The study was approved by the Beijing Tongren Hospital Ethics Committee (no. TREC2023-KY024). Patients were exempt from informed consent.

### Data collection

The following information from the electronic medical records (EMRs) of Beijing Tongren Hospital was collected: demographics (age and gender), PPIs (drug name, dosage form, administration route, usage and dosage, time of medical orders, types of medical orders, professional titles of doctors), concomitant medications (nonsteroidal anti-inflammatory drugs [NSAIDs], systemic corticosteroids, antithrombotics), grade of surgery, intraoperative blood loss, types of medical insurance, length of postoperative hospital stay, and number of drugs used.

### Evaluation criterion

The evaluation criterion was instituted to evaluate the appropriateness of perioperative prophylactic use of PPIs based on guidelines, expert consensuses, and drug instructions (American Society of Health-System Pharmacists 1999; Hospital Pharmacy Committee of Chinese Pharmaceutical Association 2020; Writing Group of Expert Consensus on the Preventive Application of Proton Pump Inhibitors 2018; Bai et al. 2018; National Health Commission of the People’s Republic of China 2020). The evaluation criterion included six aspects: indication, usage and dosage, drug selection, administration route, solvent, and duration, as shown in Table 1. The appropriateness of perioperative prophylactic use of PPIs was evaluated independently by two clinical pharmacists and then reviewed by each other. If there were any disputes, the two clinical pharmacists further discussed and reached a consensus. If there were still disputes, the clinical pharmacists’ supervisor decided the outcome.

**Table 1:** Evaluation criteria for perioperative prophylactic use of proton pump inhibitors

Item	Evaluation criteria
**Indication**	**The presence of one serious risk factor from the following:** 1. Mechanical ventilation > 48 h or on extracorporeal life support 2. Platelet count < 50,000/mm^3^ (50 × 109/L), international normalized ratio > 1.5, or partial thromboplastin time > 2.0 times the control value, or taking anticoagulant or antiplatelet drugs 3. Severe craniocerebral and cervical spinal cord injuries 4. Severe burn (adult burn area > 30%, children burn area > 15%) 5. Difficult or complex operation (operation time > 3 h) 6. Acute renal failure or renal replacement therapy or chronic liver disease or acute liver failure 7. Acute respiratory distress syndrome (ARDS) 8. Shock or persistent hypotension 9. Severe infection or sepsis 10. Cardiovascular and cerebrovascular accidents 11. Severe psychological stress **The presence of at least two potential risk factors of the following:** 1. Intensive care unit stay > 1 week 2. Duration of fecal occult blood > 3 days (excluding hemorrhoids) 3. Corticosteroid therapy (> 250 mg/day hydrocortisone or equivalent daily) 4. Combined use of nonsteroidal anti-inflammatory drugs (especially long-term or high-dose use) 5. Long-term fasting and parenteral nutrition
**Usage and dosage**	PPIs should be administered once a day, and the single dose should not exceed the following doses (omeprazole 40 mg, lansoprazole 30 mg, esomeprazole 40 mg, pantoprazole 40 mg)
**Drug selection**	Omeprazole and esomeprazole are preferred unless there is no alternative or for special reasons. For example, patients using clopidogrel should preferentially select PPIs that lack inhibition of hepatic cytochrome P450 (CYP) 2C19 enzyme, such as pantoprazole or rabeprazole
**Administration route**	A standard dose of PPIs is administered intravenously or by drip Oral administration is recommended for patients who can be taken orally; intravenous administration may be considered if the patient is unable to take medications orally (including nasal feeding) or has gastrointestinal dysfunction
**Solvent**	Select the appropriate solvent according to drug instructions
**Duration**	When the patient is stable enough to tolerate adequate enteral nutrition or has taken food, the clinical symptoms begin to improve; the drug may be gradually withdrawn

### Statistical analysis

Patients were divided into two groups based on the appropriateness of perioperative prophylactic use of PPIs. A descriptive analysis was performed on the patient’s demographics, PPIs (dosage form, time of medical orders, types of medical orders, professional titles of doctors), concomitant medications (NSAIDs, systemic corticosteroids, antithrombotics), grade of surgery, intraoperative blood loss, types of medical insurance, length of postoperative hospital stay, and number of drugs used.

For continuous variables, the Student’s *t*-test or the Mann–Whitney *U*-test was used to compare the two groups. Categorical variables were described by frequencies and percentages, and between-group differences were analyzed using the chi-square test and Fisher’s exact test if necessary. Variance inflation factor (VIF) values were calculated to measure the degree of multicollinearity among the variables that were significant in the univariate analysis (*p* < 0.1). A VIF of > 10 was considered indicative of multicollinearity and excluded from the logistic regression analysis. Based on the univariate analysis and VIF values, significant variables (*p* < 0.1) were included in the multiple logistic regression analysis to identify factors associated with the inappropriate prophylactic use of PPIs. All statistical analyses were carried out using SPSS (Version 26.0). *p*-values < 0.05 were considered statistically significant.

## Results

### Patient characteristics

**Fig. 1:**
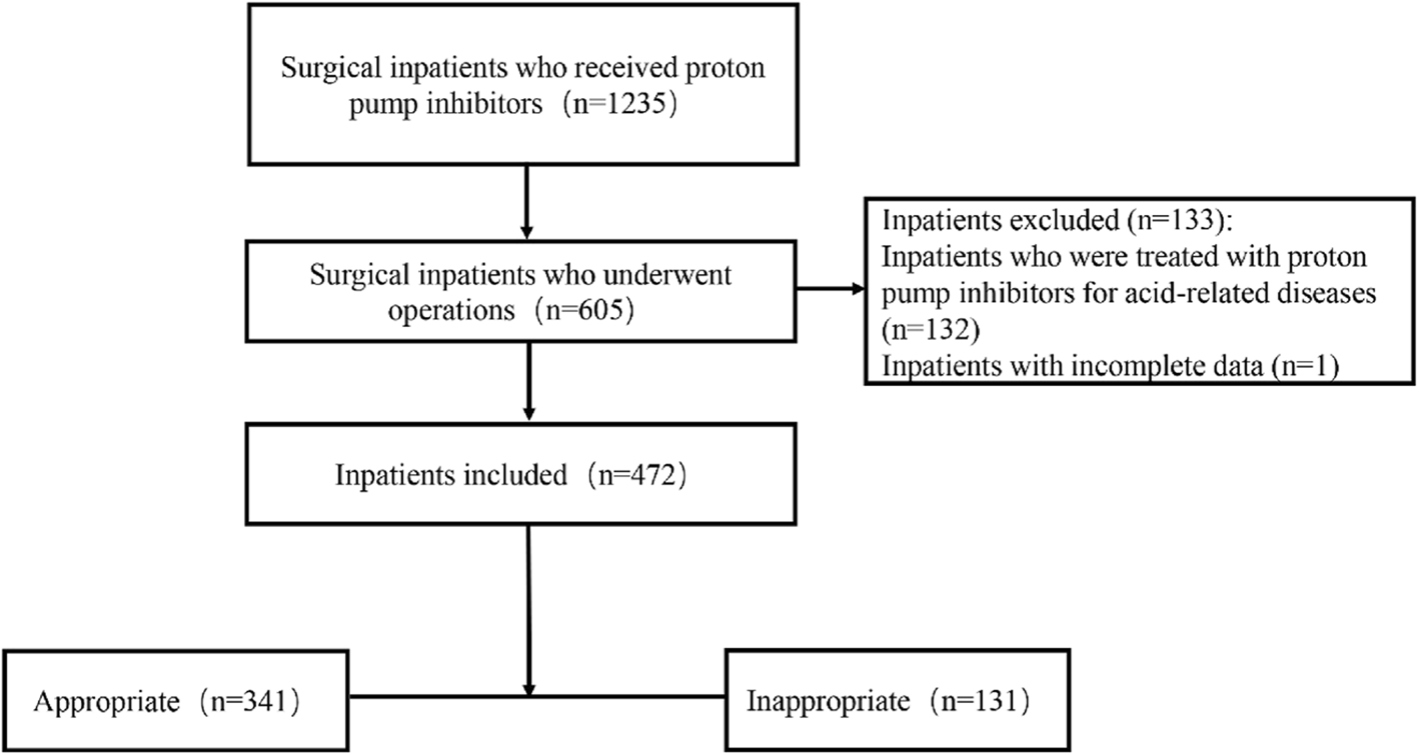
Flowchart of the patient selection process

During the study period, a total of 472 patients were finally included in our study. The procedure of patient selection is presented in Fig. 1. Among the included patients, 112 (23.7%) were over 65 years old, 328 (69.5%) were male, and 144 (30.5%) were female. The top five departments in which patients were admitted were otorhinolaryngology-head and neck surgery (58.1%), neurosurgery (10.0%), orthopedics (8.3%), thoracic surgery (7.8%), and general surgery (5.7%).

### Appropriateness of perioperative prophylactic use of PPIs

**Fig. 2:**
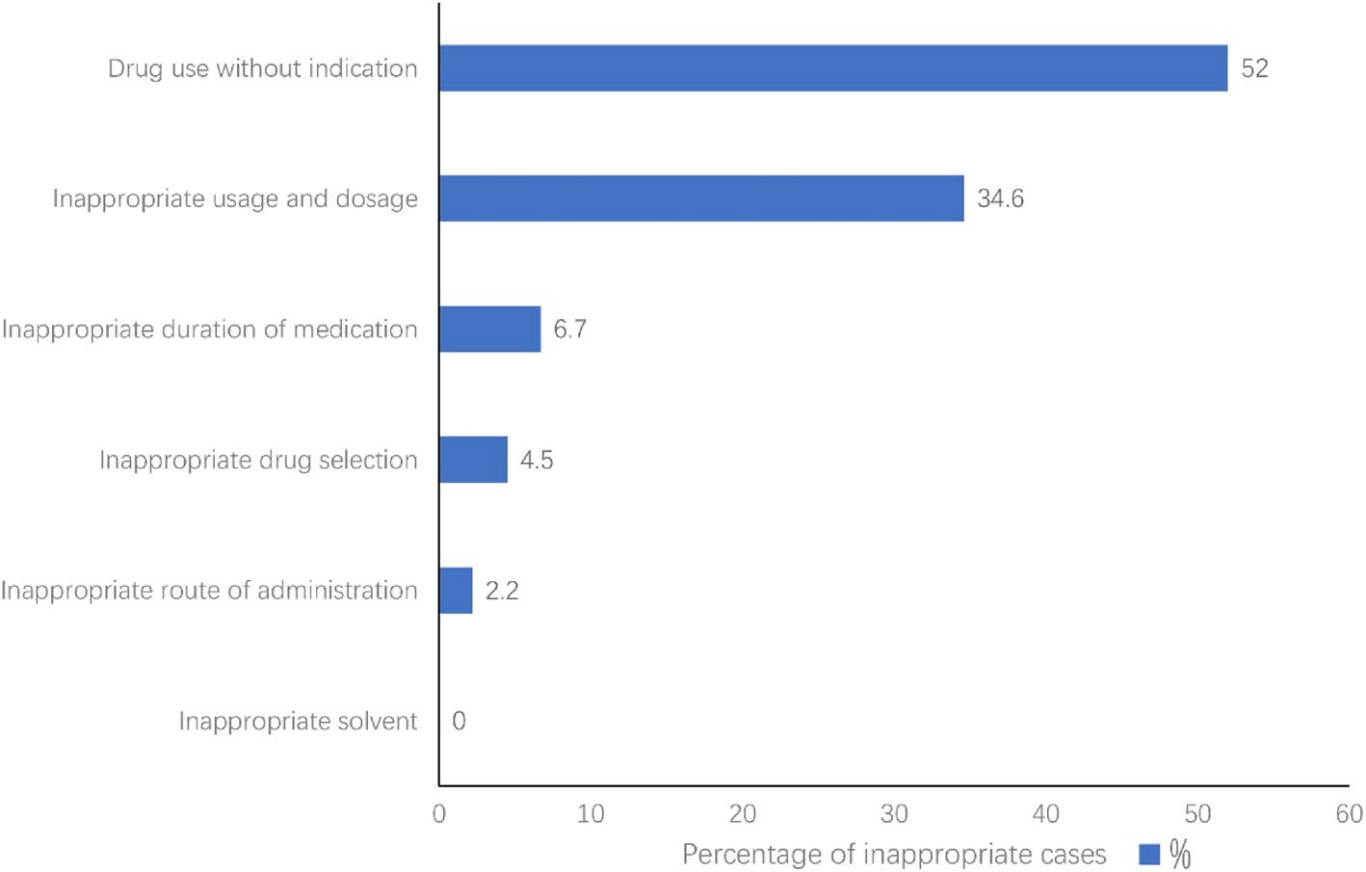
Characterization and percentages of the 179 cases of inappropriate prophylactic use of PPIs during the perioperative period

According to the evaluation criterion, the inappropriateness of perioperative prophylactic use of PPIs was found in 131 of 472 patients, with an inappropriateness rate of 27.75%. A total of 179 cases of inappropriate drug use occurred in 131 patients, of which 93 cases (52.0%) were drug use without indication, 62 cases (34.6%) were inappropriate usage and dosage, and 12 cases (6.7%) were inappropriate duration of medication (Fig. 2).

### Factors associated with the inappropriate prophylactic use of PPIs

In univariate analysis, 10 factors were significantly associated with inappropriate prophylactic use of PPIs (*p* < 0.05): gender, intraoperative blood loss, antithrombotics, systemic corticosteroids, dosage form, time of medical orders, types of medical orders, professional titles of doctors, number of drugs, and length of postoperative hospital stay (Table 2). The results of multicollinearity analysis showed that VIF values of 10 factors were less than 10. In multiple logistic regression analysis, the oral dosage form of PPIs, discharge medication of PPIs, and junior doctors were associated with more inappropriate prophylactic use of PPIs (*p* < 0.05) (Table 3). Antithrombotics and postoperative hospital stay longer than 15 days were associated with less inappropriate prophylactic use of PPIs (*p* < 0.05) (Table 3).

**Table 2:** Patient demographic and clinical characteristics

Variable	Appropriate (*n* = 341)	Inappropriate (*n* = 131)	*P*
Age (years)			0.614
≤ 65	258 (75.7)	102 (77.9)	
> 65	83 (24.3)	29 (22.1)	
Gender			0.014
Male	248 (72.7)	80 (61.1)	
Female	93 (27.3)	51 (38.9)	
Medical insurance			0.570
Yes	281 (82.4)	105 (80.2)	
No	60 (17.6)	26 (19.8)	
Intraoperative blood loss (mL)			0.002
< 1500	312 (91.5)	130 (99.2)	
≥ 1500	29 (8.5)	1 (0.8)	
Grade of surgery			0.304
1–2	18 (5.3)	4 (3.1)	
3–4	323 (94.7)	127 (96.9)	
Concomitant medications			
Antithrombotics	163 (47.8)	26 (19.8)	< 0.001
NSAIDs	65 (19.1)	29 (22.1)	0.454
Systemic corticosteroids			0.012
Low dose	200 (58.7)	83 (63.4)	
High dose	121 (35.5)	32 (24.4)	
PPIs-related information			
Dosage form			< 0.001
Injectable	326 (95.6)	44 (33.6)	
Oral	15 (4.4)	87 (66.4)	
Time of medical orders			< 0.001
Workday	341 (100)	115 (87.8)	
Weekend	0 (0.0)	16 (12.2)	
Types of medical orders			< 0.001
Standing	316 (92.7)	59 (45.0)	
Statim	24 (7.0)	24 (18.3)	
Discharge	1 (0.3)	48 (36.6)	
Professional titles of doctors			< 0.001
Junior	24 (7.0)	34 (26.0)	
Middle	157 (46.0)	48 (36.6)	
Senior	160 (46.9)	49 (37.4)	
Number of drugs			< 0.001
≤ 10	85 (24.9)	76 (58.0)	
11–20	178 (52.2)	46 (35.1)	
> 20	78 (22.9)	9 (6.9)	
Length of postoperative hospital stay (days)			< 0.001
≤ 7	89 (26.1)	93 (71.0)	
8–15	156 (45.7)	29 (22.1)	
> 15	96 (28.2)	9 (6.9)	

*NSAIDs* Nonsteroidal anti-inflammatory drugs

**Table 3:** Multiple logistic regression analysis of factors associated with the inappropriate prophylactic use of PPIs during the perioperative period

Variable	Assignment	Adjusted OR (95% *CI*)	*P*
Gender	Male = 0, female = 1	1.256 (0.649–2.430)	0.499
Age	≤ 65 = 0, > 65 = 1	1.098 (0.498–2.420)	0.817
Intraoperative blood loss	< 1500 = 0, ≥ 1500 = 1	0.242 (0.027, 2.128)	0.201
Antithrombotics	No = 0, yes = 1	0.313 (0.136–0.721)	0.006
Systemic corticosteroids	No = 0		
	Low dose = 1	1.253 (0.366–4.295)	0.720
	High dose = 2	1.049 (0.278–3.961)	0.944
Dosage form	Injectable = 0, oral = 1	18.301 (7.497–44.671)	< 0.001
Time of medical orders	Workday = 0, weekend = 1		0.998
Types of medical orders	Standing = 0		
	Statim = 1	1.867 (0.780–4.470)	0.161
	discharge = 2	11.739 (1.289–106.886)	0.029
Professional titles of doctors	Senior = 0		
	Middle = 1	1.477 (0.725–3.008)	0.283
	Junior = 2	9.167 (3.459–24.299)	< 0.001
Number of drugs	≤ 10 = 0		
	11–20 = 1	0.605 (0.299–1.224)	0.162
	> 20 = 2	0.818 (0.220–3.043)	0.764
Length of postoperative hospital stay	≤ 7 = 0		
	8–15 = 1	0.515 (0.252–1.056)	0.070
	> 15 = 2	0.262 (0.072–0.951)	0.042

## Discussion

To the best of our knowledge, this is the first study to explore the prevalence and characteristics of inappropriate prophylactic use of PPIs during the perioperative period and its associated factors. In our study, the inappropriateness rate of perioperative prophylactic use of PPIs was 27.75%, which was lower than in previous studies (Chen et al. 2022; Zhang et al. 2021; Bez et al. 2013). Different departments, study population size, different evaluation criteria, and study duration may lead to different inappropriateness rates (Liu et al. 2021). For example, previous studies had focused on patients admitted to specific surgical departments; however, our research was focused on patients in the perioperative period, which was also our biggest highlight. Meanwhile, the underprescription of PPIs was not considered in our study because our study population was limited to patients who had already used PPIs to prevent perioperative SRMD. In addition, we had taken some measures to improve the rationality of perioperative prophylactic use of PPIs prior to the study.

In our study, the most common problem was drug use without indication, which was also a major problem faced by other studies (Ali et al. 2019; Chen et al. 2022; Bez et al. 2013). Owing to concern about the risk of perioperative gastrointestinal bleeding, clinicians often prescribe PPIs to patients. In fact, the risk of perioperative gastrointestinal bleeding is only 4%, so there is no need to prescribe PPIs to prevent SRMD in low-risk perioperative patients (Li et al. 2022). Superior efficacy for acid suppression and the availability of generic formulations have led prescribers to favor the use of PPIs. This has also led to the overuse and abuse of PPIs (Savarino et al. 2018). A total of 25–70% of patients lack indications for PPIs use, resulting in unnecessary expenditure of close to £2 billion each year (Forgacs and Loganayagam 2008). In addition, overuse and abuse of PPIs are associated with a variety of adverse events in patients, such as acute kidney injury, *Clostridioides difficile* infection, pneumonia, and bone fractures (Savarino et al. 2017). Therefore, PPIs should only be used in patients with risk factors for SRMD during the perioperative period based on domestic and foreign guidelines and expert recommendations (American Society of Health-System Pharmacists 1999; Hospital Pharmacy Committee of Chinese Pharmaceutical Association 2020; Writing Group of Expert Consensus on the Preventive Application of Proton Pump Inhibitors 2018; Bai et al. 2018; National Health Commission of the People’s Republic of China 2020). Criteria for appropriate use include one serious risk factor or two potential risk factors, as shown in Table 1.

In this study, inappropriate usage and dosage were mainly reflected in the frequency of medication. The current literature showed that there was a contradiction in the frequency of administration, which focused on once or twice a day (American Society of Health-System Pharmacists 1999; Hospital Pharmacy Committee of Chinese Pharmaceutical Association 2020; Writing Group of Expert Consensus on the Preventive Application of Proton Pump Inhibitors 2018; Bai et al. 2018; National Health Commission of the People’s Republic of China 2020). PPIs maintain a longer-lasting acid inhibition, which is due to the fact that the proton pump cannot be recovered once it is deactivated, and its acid secretion can only be restored after the formation of a new proton pump. From the perspective of pharmacodynamics, the acid inhibition effect of PPIs can be maintained for 16–18 h (Savarino et al. 2018). Based on the above considerations, PPIs were administered once a day as an evaluation criterion in this study.

There is no clear standard for the duration of PPIs, but it is suggested that if patients can tolerate adequate enteral nutrition or have taken food, the clinical symptoms begin to improve as indications for drug withdrawal. Our study showed that 12 (9.2%) patients had an inappropriate duration of medication, which was mainly reflected in patients who continued to use PPIs even after discharge. Although the proportion of this problem is small, we should pay attention to it. Long-term use of PPIs may increase the risk of adverse reactions, especially in the absence of guidance from doctors or pharmacists after discharge. In our study, 78.4% of patients received intravenous PPIs, but only 3.1% of patients had a problem with the inappropriate route of administration, which was significantly lower than in previous studies (Li et al. 2022). This was attributed to the fact that patients admitted to otorhinolaryngology-head and neck surgery, neurosurgery, thoracic surgery, and other departments could not take medication orally or had gastrointestinal dysfunction after surgery.

Our study showed that oral dosage form of PPIs, discharge medication of PPIs, and junior doctors were associated with an increased risk of inappropriate prophylactic use of PPIs during the perioperative period, which was another highlight of this study. To our surprise, up to 87 of the 102 patients who received oral dosage form of PPIs had a problem with inappropriate prophylactic use of PPIs. In response to this study’s findings, a number of changes will be implemented in our hospital to address inappropriate use of PPIs for SRMD prophylaxis. Clinical pharmacists will strengthen training for doctors on the oral dosage form of PPIs. In particular, we recommend the prescriber to evaluate the need for ongoing PPI therapy at the time of the switch from injectable to oral and discontinue the PPI if it is no longer needed. Of the 87 patients who received inappropriate oral PPI orders, 48 were due to inappropriate discharge medication. It should be given more attention that discharge prescriptions make up 36.6% of the inappropriate orders. This result again confirms that inappropriate PPIs continuation upon discharge is a common issue despite several guidelines and ongoing global attention in recent years to highlight the risks versus benefit. The inappropriate PPIs continuation upon discharge exposes patients to excess risk of adverse events. Clinical pharmacists will rigorously review the surgical discharge medication of PPIs. In teaching hospital settings, it is often the primary responsibility of the junior doctor to enter medication orders after reviewing the case with the attending doctor. We will continue to explore the reasons behind the association between junior doctors and inappropriate prophylactic use of PPIs. In view of the current result, in addition to the training and education of doctors, especially junior doctors, adding a reminder function to the doctor’s order system may be a good approach (Clarke et al. 2021; Fan et al. 2023). When surgeons prescribe PPIs, the system will automatically pop up a reminder interface, including the indication of PPIs, usage and dosage, and the course of treatment, which will help surgeons use PPIs rationally.

The concomitant use of antithrombotics was associated with a decreased risk of inappropriate prophylactic use of PPIs during the perioperative period. Eid also found a similar conclusion that the combination of anticoagulants had a protective effect on the rational use of PPIs (Eid et al. 2010). However, another study showed that the concomitant use of aspirin or anticoagulants promoted inappropriate stress ulcer prophylaxis (Issa et al. 2012). Other studies had found that glucocorticoids were associated with the irrational use of PPIs, but our study did not find a similar conclusion (Li et al. 2022; Schepisi et al. 2016). The inappropriate prophylactic use of PPIs was reduced as the length of postoperative hospital stay (longer than 15 days) lengthened. This may be due to the increased risk factors for prophylactic use of PPIs in patients with prolonged hospital stay after surgery. Previous studies had explored the relationship between the length of hospital stay and the irrational use of PPIs, which was different from our study (Li et al. 2022; Mayet 2007).

Our study has the following limitations. First, this was a retrospective, single-center study. However, we believe our result is representative based on our hospital scale. Second, the study population size was small. The outbreak of the novel coronavirus reduced the number of inpatients during the study period. Third, there was a large percentage of otorhinolaryngology-head and neck surgery patients, which might not be representative of surgical patients in general. Finally, our study did not assess the underprescription of PPIs and track patient comorbidities and outcomes such as bleeding or other adverse events.

## Conclusions

Our study demonstrates that the inappropriate prophylactic use of PPIs during the perioperative period is common in China. The most common problem is drug use without indication. Additionally, oral dosage form of PPIs, discharge medication of PPIs, and junior doctors are associated with more frequent inappropriate prophylactic use of PPIs. Consequently, regulation authorities and pharmacists should take more targeted measures to promote the rational prophylactic use of PPIs during the perioperative period.

### Supplementary Information

**Additional file 1.** The evaluation criterion.

## Data Availability

The datasets used and analyzed during the current study are available from the corresponding author on reasonable request.

## Abbreviations

EMRs: Electronic medical records
H_2_RAs: Histamine-2 receptor antagonists
NSAIDs: Nonsteroidal anti-inflammatory drugs
PPIs: Proton pump inhibitors
SRMD: Stress-related mucosal disease
VIF: Variance inflation factor
