# Supplementary material for: Analysis of inappropriate prophylactic use of proton pump inhibitors during the perioperative period: an observational study
Source: Perioper Med (Lond). 2024 Mar 14;13:19. doi: 10.1186/s13741-024-00376-w (PMC10938722; doi:10.1186/s13741-024-00376-w)
Supplement: Supplementary file 1 — Additional file 1. The evaluation criterion. [file 13741_2024_376_MOESM1_ESM.docx]

**Sources of the evaluation criterion**

1. American Society of Health-System Pharmacists. ASHP therapeutic guidelines on stress ulcer prophylaxis. Am J Health Syst Pharm. 1999; 56:347-79.

Recommendation: Risk factors for ICU patients have been delineated in trials comparing prophylaxis with no prophylaxis by using clinically important bleeding as an endpoint. Prophylaxis is recommended in patients with coagulopathy or patients requiring mechanical ventilation for more than 48 hours. (Strength of evidence = C) Prophylaxis is also recommended in patients with a history of GI ulceration or bleeding within one year before admission and in patients with at least two of the following risk factors: sepsis, ICU stay of more than one week, occult bleeding lasting six days or more, and use of high-dose corticosteroids (>250 mg per day of hydrocortisone or the equivalent). (Strength of evidence = D) Recommendations for specific prophylactic medications can be found in the Medications used for prophylaxis section.

Recommendation: Prophylaxis is recommended for ICU patients with a Glasgow Coma Score of ≤10 (or the inability to obey simple commands) or thermal injuries to >35% of their BSA. (Strength of evidence = B) ICU patients with partial hepatectomy may also benefit from prophylaxis. (Strength of evidence = C) Prophylaxis may also be indicated in ICU patients with multiple trauma (e.g., Injury Severity Score of ≥16), transplantation patients in the ICU perioperatively, ICU patients with hepatic failure, and ICU patients with spinal cord injuries. (Strength of evidence = D)

Recommendation (adults): Stress ulcer prophylaxis is not recommended for adult patients in non-ICU settings. (Strength of evidence = B for general medical and surgical patients with fewer than two risk factors for clinically important bleeding; strength of evidence = D for patients with two or more risk factors)

2. Hospital Pharmacy Committee of Chinese Pharmaceutical Association. Expert consensus on optimal application of proton pump inhibitors. Chin J Hosp Pharm. 2020; 40(21):2195-213.

-PPIs for the prevention of stress ulcers in critically ill patients is only applicable to high-risk groups, and the risk factors are detailed in the literature.

-For patients at high risk for stress ulcers, regular doses of PPIs should be administered intravenously or by drip after risk factors appear. When the patient’s condition is stable and can tolerate sufficient enteral nutrition or has eaten, clinical symptoms begin to improve, or transferred to the general ward can be changed to oral or gradual withdrawal of medication.

3. Writing Group of Expert Consensus on the Preventive Application of Proton Pump Inhibitors. Expert consensus on the preventive application of proton pump inhibitors (2018). Journal of Chinese physician. 2018; 20(12):1775-80.

- Patients who receive mechanical ventilation and have a high risk of bleeding (including those who are expected to receive mechanical ventilation for more than 48 hours or have abnormal coagulation function) need to use PPIs.

- Patients admitted to the ICU who have one of the following risk factors may use PPIs. (1) severe trauma; (2) acute renal failure; (3) Acute liver failure; (4) Acute lung injury; (5) Respiratory failure or coagulation dysfunction.

- PPIs may also be used in patients with two or more of the following risk factors. (1) Sepsis; (2) ICU stay longer than 1 week; (3) Duration of fecal occult blood ≥ 3days; (4) Use of high doses of glucocorticoids (>250 mg/d hydrocortisone or equivalent daily).

- PPIs can be used in patients with one of the following risk factors if they have severe psychological stress such as psychological trauma, excessive stress and other stressors. The risk factors are detailed in the literature.

- For the prevention of stress ulcers in high-risk patients, the regular dose of PPIs can be used once a day, and intravenous administration can only be considered for those who cannot be given orally.

4. Bai Y, Li Y, Ren X, Li Z. Expert advice on the prevention and treatment of stress ulcer (2018). Natl Med J China. 2018; 98(42):3392-4.

- For critically ill patients, prophylactic drugs should be used if they have one of the following high-risk factors (see literature). If they also have any two of the following risk factors (see literature), prophylactic drugs should also be considered.

- PPI is the preferred medication for preventing stress ulcer, and it is recommended to administer standard dose intravenous infusion twice a day for at least 3 days after the onset of the primary disease.
